# Supplementary material for: The STRIPAK signaling complex regulates dephosphorylation of GUL1, an RNA-binding protein that shuttles on endosomes
Source: PLoS Genet. 2020 Sep 30;16(9):e1008819. doi: 10.1371/journal.pgen.1008819 (PMC7550108; doi:10.1371/journal.pgen.1008819)
Supplement: S1 Fig — (A) In total 4,349 proteins were quantified in this study, compared to 4,193 in our previous study, 93% of which we were covered in this study. (B) The commonly used deletion strain Δpro11 was used to compare the quantification between the two analyses and a Pearson’s correlation coefficient of 0.7339 was calculated. (PDF) [file pgen.1008819.s001.pdf]

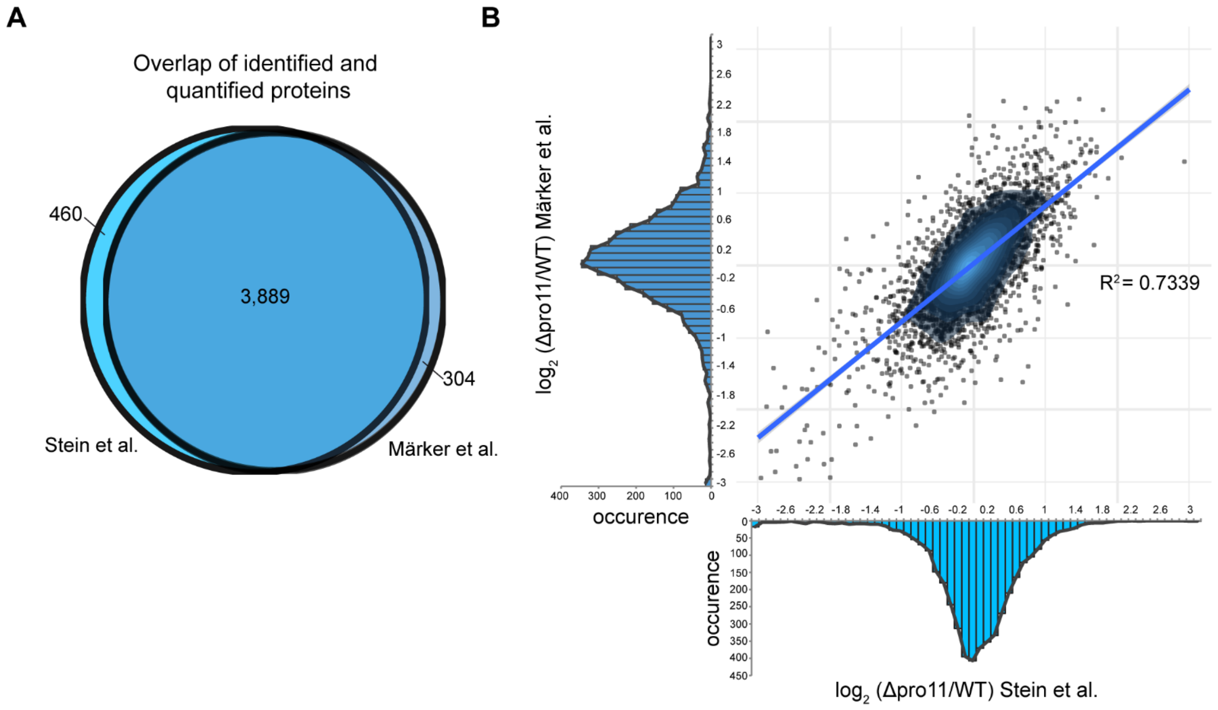

S1 Fig. Proteins identified and quantified in this and the previous study [1]. (A) In total 4,349 proteins were quantified in this study, compared to 4,193 in our previous study, 93 % of which we were covered in this study. (B) The commonly used deletion strain Δpro11 was used to compare the quantification between the two analyses and a Pearson's correlation coefficient of 0.7339 was calculated.

1. Märker R, Blank-Landeshammer B, Beier-Rosberger A, Sickmann A, Kück U. Phosphoproteomic analysis of STRIPAK mutants identifies a conserved serine phosphorylation site in PAK kinase CLA4 to be important in fungal sexual development and polarized growth. Mol Microbiol. 2020.
