# Supplementary material for: The STRIPAK signaling complex regulates dephosphorylation of GUL1, an RNA-binding protein that shuttles on endosomes
Source: PLoS Genet. 2020 Sep 30;16(9):e1008819. doi: 10.1371/journal.pgen.1008819 (PMC7550108; doi:10.1371/journal.pgen.1008819)
Supplement: S2 Fig — (A, C) In total 9,773 phosphopeptides originating from 2,465 proteins were quantified in this study, compared to 10,635 phosphopeptides from 2,489 phosphoproteins in the previous study [21], 58% and 84% of which were commonly identified, respectively. (B) The deletion strain Δpro11 was used to compare the quantification between the two analyses and a Pearson’s correlation coefficient of 0.621 was calculated for the commonly identified phosphopeptides. (PDF) [file pgen.1008819.s002.pdf]

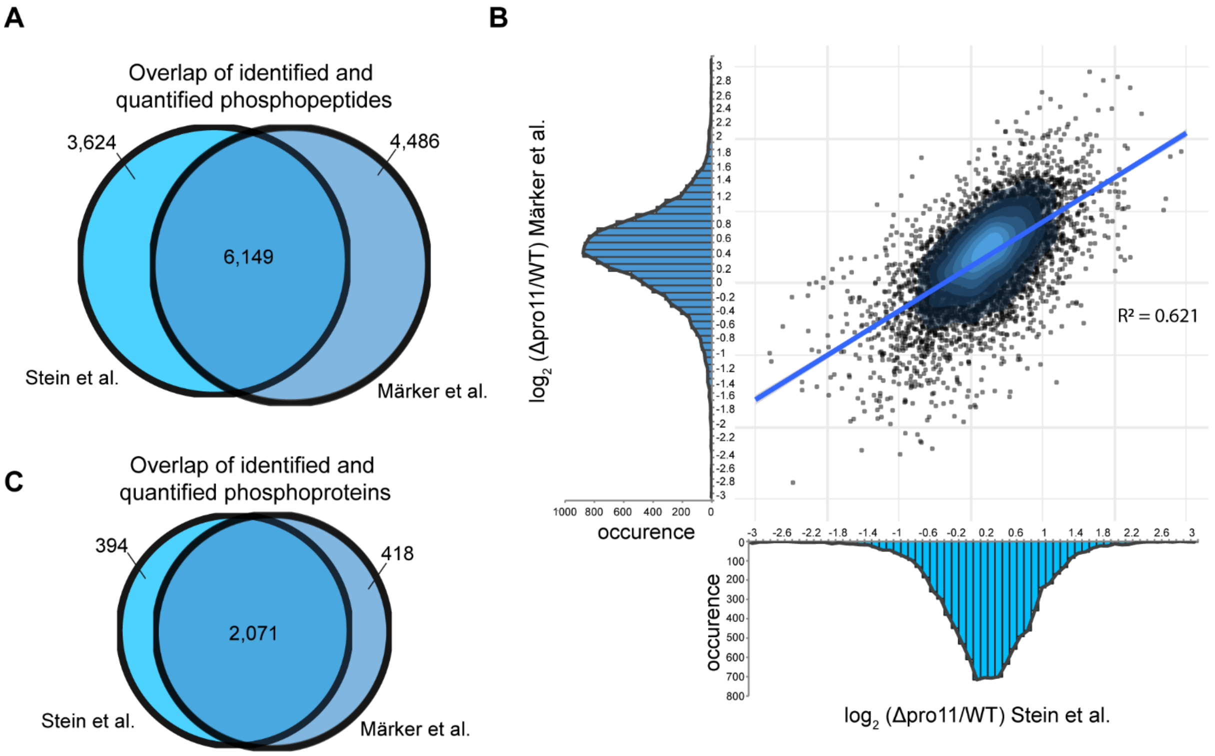

S2 Fig. Phosphoproteins and –peptides identified and quantified in this and the previous study [1]. (A, C) In total 9,773 phosphopeptides originating from 2,465 proteins were quantified in this study, compared to 10,635 phosphopeptides from 2,489 phosphoproteins in the previous study [1], 58 % and 84 % of which were commonly identified, respectively. (B) The deletion strain Δpro11 was used to compare the quantification between the two analyses and a Pearson's correlation coefficient of 0.621 was calculated for the commonly identified phosphopeptides.

1. Märker R, Blank-Landeshammer B, Beier-Rosberger A, Sickmann A, Kück U. Phosphoproteomic analysis of STRIPAK mutants identifies a conserved serine phosphorylation site in PAK kinase CLA4 to be important in fungal sexual development and polarized growth. Mol Microbiol. 2020.
