# Supplementary material for: The STRIPAK signaling complex regulates dephosphorylation of GUL1, an RNA-binding protein that shuttles on endosomes
Source: PLoS Genet. 2020 Sep 30;16(9):e1008819. doi: 10.1371/journal.pgen.1008819 (PMC7550108; doi:10.1371/journal.pgen.1008819)
Supplement: S3 Fig — Strains were grown on MMS and cellophane for four days. Wild type served as control. Dotted lines indicate the hyphal area of microscopic images. Not drawn to scale. (PDF) [file pgen.1008819.s003.pdf]

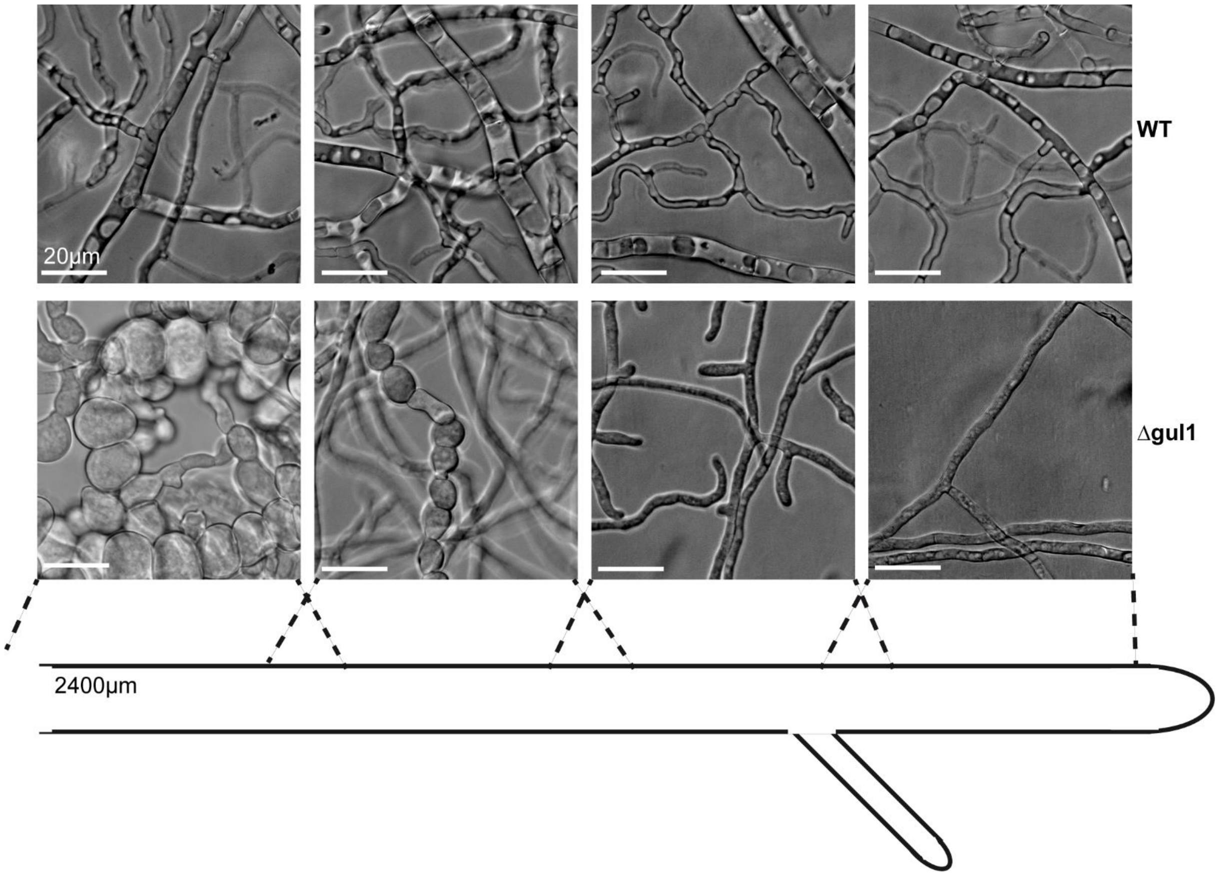

S3 Fig. Phenotype of ∆gul1 hyphae in different regions of the colony. Strains were grown on MMS and cellophane for four days. Wild type served as control. Dotted lines indicate the hyphal area of microscopic images. Not drawn to scale.
