# Supplementary material for: The STRIPAK signaling complex regulates dephosphorylation of GUL1, an RNA-binding protein that shuttles on endosomes
Source: PLoS Genet. 2020 Sep 30;16(9):e1008819. doi: 10.1371/journal.pgen.1008819 (PMC7550108; doi:10.1371/journal.pgen.1008819)
Supplement: S4 Fig — Strains were grown for 3 days in liquid media (BMM) as a surface culture. For each strain, 10 μg of crude protein extract were subjected to SDS-PAGE. Western blot analysis was performed with an anti-GFP antibody and an anti- α-Tubulin antibody as control. GUL1 tagged with GFP has a mass of 175 kDa, while α-Tubulin has a mass of 55 kDa. GUL1-GFP was detected in all six different phospho-mutants (S180A and S180E, S216A, S216E, S1343A and S1343E). Wild type and a complemented Δgul1 strain were used as control. (PDF) [file pgen.1008819.s004.pdf]

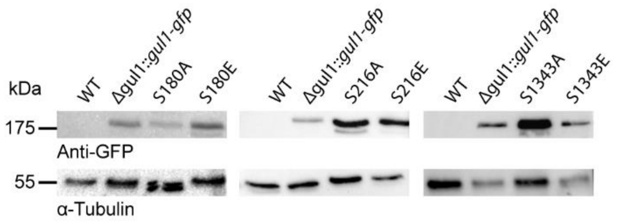

S4 Fig. Expression control of phospho-mutated variants of GUL1 tagged with GFP. Strains were grown for 3 days in liquid media (BMM) as a surface culture. For each strain, 10 µg of crude protein extract were subjected to SDS-PAGE. Western blot analysis was performed with an anti-GFP antibody and an anti- α-Tubulin antibody as control. GUL1 tagged with GFP has a mass of 175 kDa, while α-Tubulin has a mass of 55 kDa. GUL1-GFP was detected in all six different phospho-mutants (S180A and S180E, S216A, S216E, S1343A and S1343E). Wild type and a complemented Δgul1 strain were used as control.
