# Supplementary material for: The STRIPAK signaling complex regulates dephosphorylation of GUL1, an RNA-binding protein that shuttles on endosomes
Source: PLoS Genet. 2020 Sep 30;16(9):e1008819. doi: 10.1371/journal.pgen.1008819 (PMC7550108; doi:10.1371/journal.pgen.1008819)
Supplement: S5 Fig — Examples of kymographs, used for the analysis of moving GUL1. For this analysis, kymographs were generated for a distance of 50 μm 20 μm beyond the hyphal tip. The shuttling of GUL1-GFP was measured in at least 15 different hyphae per strain (S3 and S4 Datasets). (PDF) [file pgen.1008819.s005.pdf]

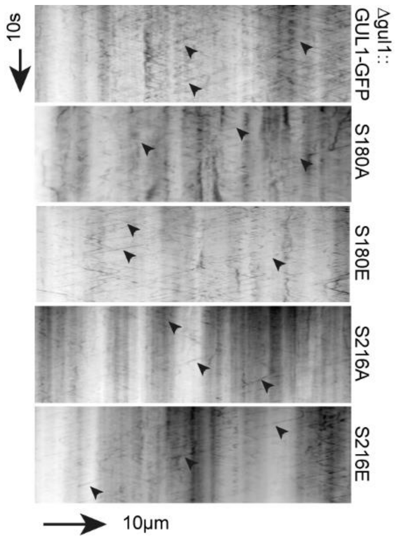

S5 Fig. Shuttling signals of GUL1-GFP phospho-variants. Examples of kymographs, used for the analysis of moving GUL1. For this analysis, kymographs were generated for a distance of 50 µm 20 µm beyond the hyphal tip. The shuttling of GUL1-GFP was measured in at least 15 different hyphae per strain (S3-S4 dataset).
