# Supplementary material for: The STRIPAK signaling complex regulates dephosphorylation of GUL1, an RNA-binding protein that shuttles on endosomes
Source: PLoS Genet. 2020 Sep 30;16(9):e1008819. doi: 10.1371/journal.pgen.1008819 (PMC7550108; doi:10.1371/journal.pgen.1008819)
Supplement: S6 Fig — (A) Example of a kymograph, used for the analysis of moving GUL1 in a deletion strain of pro45. Kymograph was generated for a distance of 100 μm 20 μm beyond the hyphal tip. (B) The shuttling of GUL1-GFP was measured in 7 different hyphae per strain in triplicates with at least 18 moving particles per hyphae (S10 Movie, S3 and S4 Datasets). (PDF) [file pgen.1008819.s006.pdf]

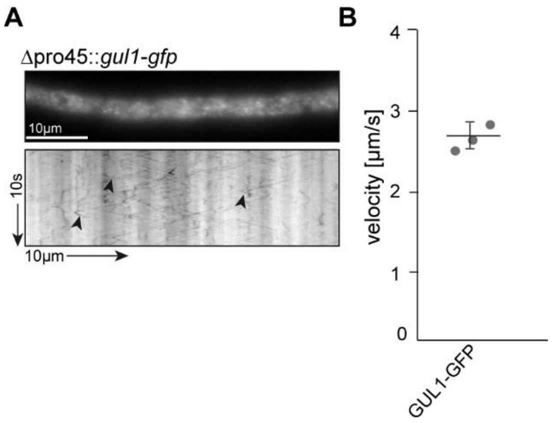

S6 Fig. Shuttling of GUL1-GFP in Δpro45. (A) Example of a kymograph, used for the analysis of moving GUL1 in a deletion strain of pro45. Kymograph was generated for a distance of 100 µm 20 µm beyond the hyphal tip. (B) The shuttling of GUL1-GFP was measured in 7 different hyphae per strain in triplicates with at least 18 moving particles per hyphae (S4 movie, S3-S4 dataset).
