# Supplementary material for: The STRIPAK signaling complex regulates dephosphorylation of GUL1, an RNA-binding protein that shuttles on endosomes
Source: PLoS Genet. 2020 Sep 30;16(9):e1008819. doi: 10.1371/journal.pgen.1008819 (PMC7550108; doi:10.1371/journal.pgen.1008819)
Supplement: S7 Fig — Y-ions are depicted in blue, b-ions in red, b-ions with neutral loss of H3PO4 in pink and iTRAQ reporter ions in purple. B- and y ions were used for scoring by the Mascot search algorithm, while all ions were used by the phosphoRS algorithm [77] to calculate the phosphorylation site probability of 99.6% for this peptide The b3-P, b4-P and and y12 ions are indicative of the phosphorylation on serine 3. (PDF) [file pgen.1008819.s007.pdf]

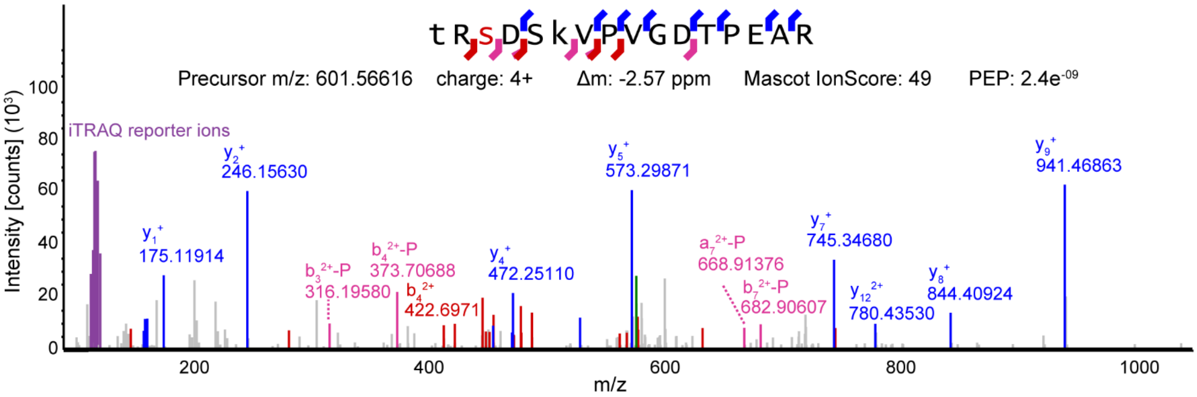

S7 Fig. Example for a tandem mass spectrum of the peptide TRSDSKVPVGDTPEAR, identifying phosphorylation of GUL1 residue S1289. Y-ions are depicted in blue, b-ions in red, b-ions with neutral loss of H_3PO_4 in pink and iTRAQ reporter ions in purple. B- and y ions were used for scoring by the Mascot search algorithm, while all ions were used by the phosphoRS algorithm [1] to calculate the phosphorylation site probability of 99.6 % for this peptide The b_3-P, b_4-P and and y_{12} ions are indicative of the phosphorylation on serine 3.

1. Taus T, Köcher T, Pichler P, Paschke C, Schmidt A, Henrich C, et al. Universal and confident phosphorylation site localization using phosphoRS. J Proteome Res. 2011;10(12):5354-62.
