# Supplementary material for: The STRIPAK signaling complex regulates dephosphorylation of GUL1, an RNA-binding protein that shuttles on endosomes
Source: PLoS Genet. 2020 Sep 30;16(9):e1008819. doi: 10.1371/journal.pgen.1008819 (PMC7550108; doi:10.1371/journal.pgen.1008819)
Supplement: S8 Fig — (A) Genomic situation of the wild type and Δgul1. Genes are indicated by arrows showing primers for the verification of the deletion via PCR fragments, which are shown as grey lines. The thick grey line indicate DNA fragments used as probes for Southern hybridization. The restriction sites of the enzyme HindIII are displayed, which was used for restriction of the DNA for Southern blot analysis. Dotted lines display areas for homologous integration. Not drawn to scale (B) PCR analysis for the verification of the gul1 deletion. Integration of 5’-flank gul1, 3’-flank gul1 and gul1 was tested. Genomic DNA of the wild type (wt) served as control. Negative control (NK) contained no DNA. (C) Autoradiograph of Southern blot hybridization with radioactively labeled probes specific for gul1 and hph after digestion of the genomic DNA of wt and the gul1 deletion strain with HindIII. (PDF) [file pgen.1008819.s008.pdf]

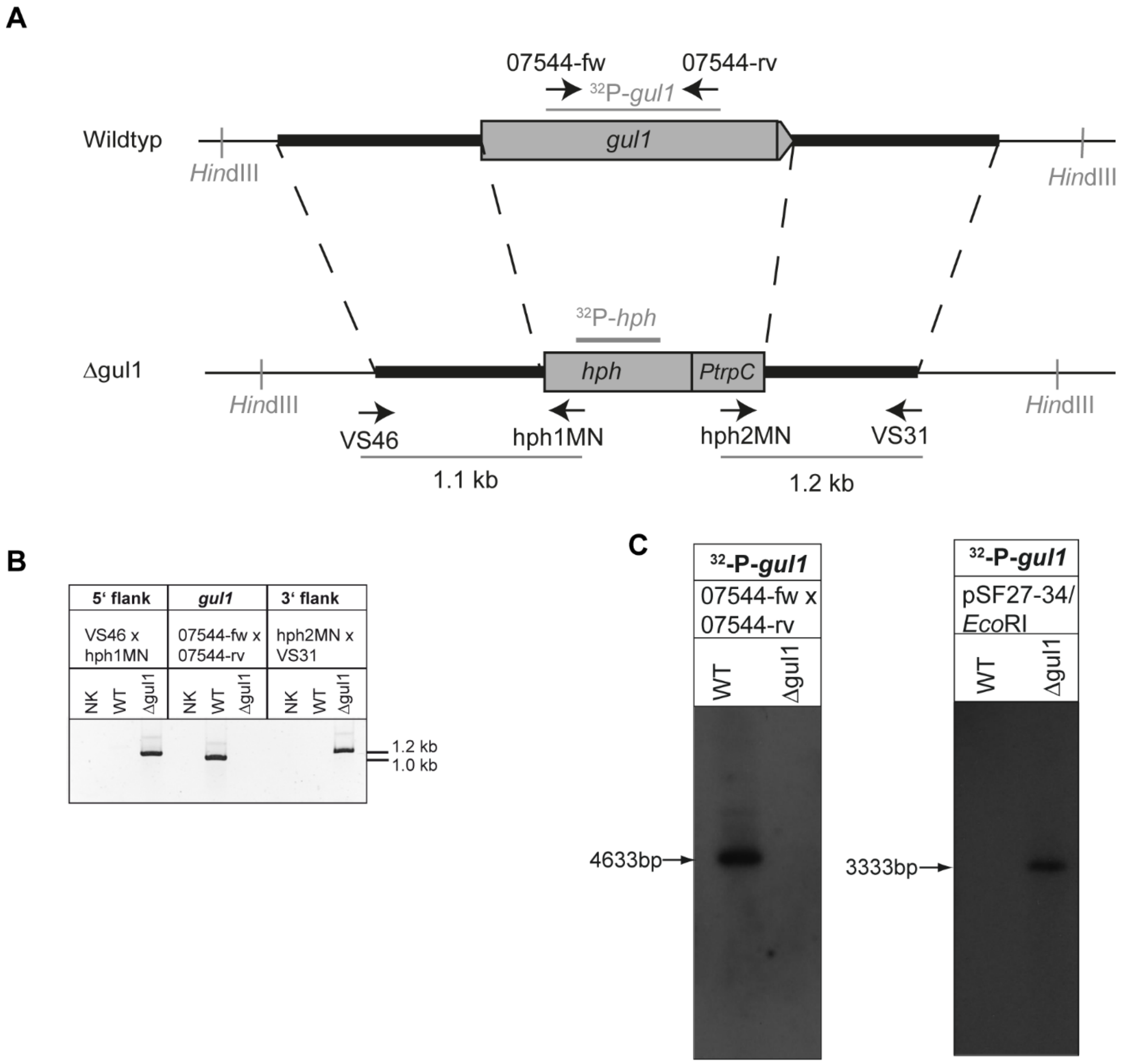

S8 Fig. Deletion strategy and verification of a *gul1* deletion strain at the *gul1* locus via PCR and Southern blot analysis. (A) Genomic situation of the wild type and Δgul1. Genes are indicated by arrows showing primers for the verification of the deletion via PCR fragments, which are shown as grey lines. The thick grey line indicate DNA fragments used as probes for Southern hybridization. The restriction sites of the enzyme *Hin*dIII are displayed, which was used for restriction of the DNA for Southern blot analysis. Dotted lines display areas for homologous integration. Not drawn to scale (B) PCR analysis for the verification of the *gul1* deletion. Integration of 5'-flank *gul1*, 3'-flank *gul1* and *gul1* was tested. Genomic DNA of the wild type (wt) served as control. Negative control (NK) contained no DNA. (C) Autoradiograph of Southern blot hybridization with radioactively labeled probes specific for *gul1* and *hph* after digestion of the genomic DNA of wt and the *gul1* deletion strain with *Hin*dIII.
