# Supplementary material for: The STRIPAK signaling complex regulates dephosphorylation of GUL1, an RNA-binding protein that shuttles on endosomes
Source: PLoS Genet. 2020 Sep 30;16(9):e1008819. doi: 10.1371/journal.pgen.1008819 (PMC7550108; doi:10.1371/journal.pgen.1008819)
Supplement: S9 Fig — (A) Genomic situation of the wt, Δgul1 and Δpro45. Arrows indicate primers for the verification of the deletion via PCR, which are shown as black lines. The thick grey lines indicate DNA fragments used as probe for Southern hybridization. The restriction sites of the enzymes are displayed, which were used for restriction of the DNA for Southern blot analysis. Dotted lines display areas for homologous integration. (B) PCR analysis for the verification of the gul1- and pro45 deletion. Integration of 5’-flank gul1, 3’-flank gul1 and gul1 was tested, as well as 5’-flank pro45, 3’-flank pro45 and pro45 in S156228. Genomic DNA of the wt served as control. Negative control (NK) contained no DNA. (C) Autoradiograph of Southern blot hybridization with radioactively labeled probes specific for hph, gul1 and pro45. Genomic DNA for hybridization with gul1, pro45 and hph was digested with HindIII, EcoRI and PvuII, respectively. (PDF) [file pgen.1008819.s009.pdf]

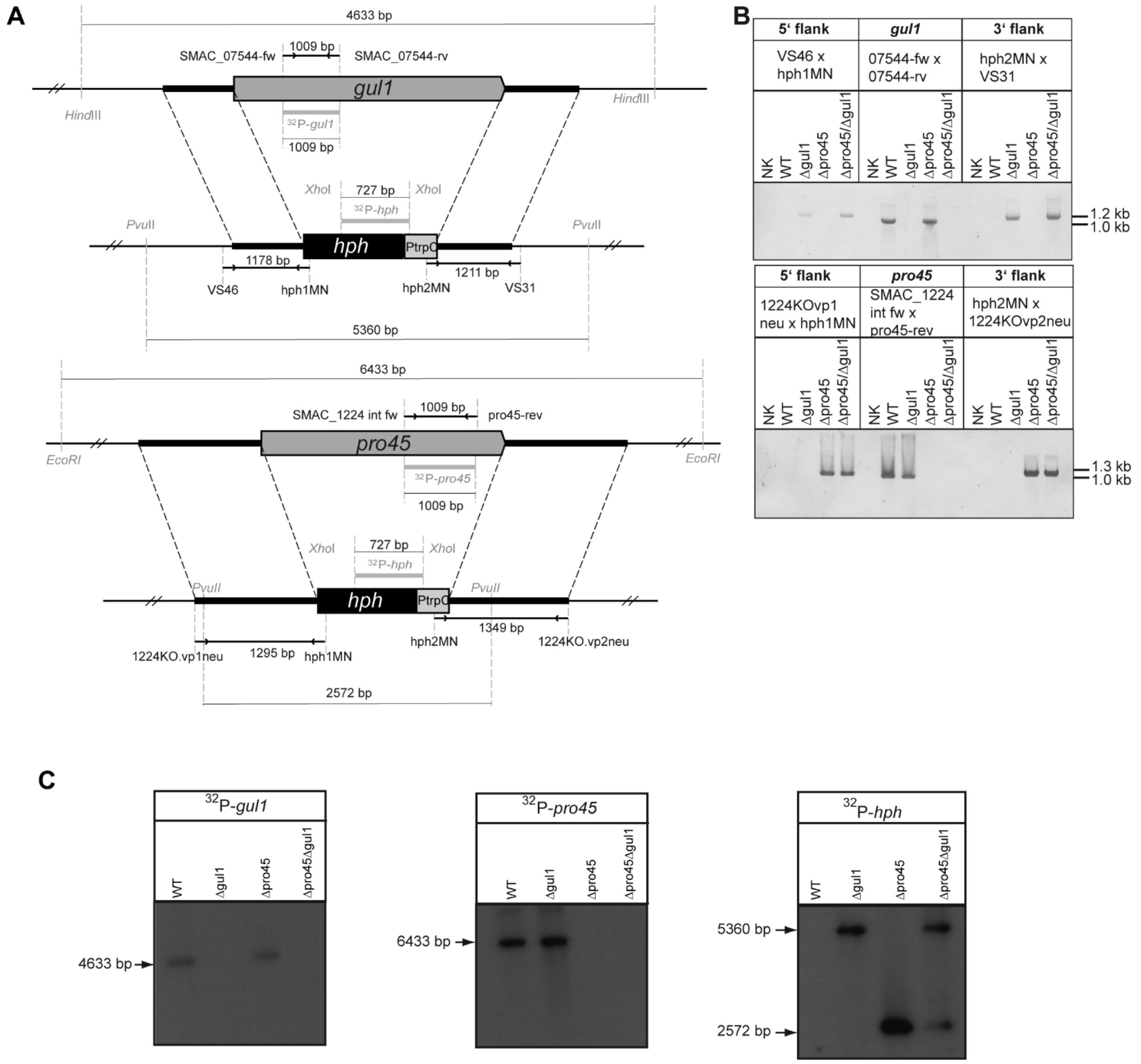

S9 Fig. Deletion strategy and verification of double deletion of *gul1* and *pro45* via PCR and Southern blot analysis. (A) Genomic situation of the wt, Δgul1 and Δpro45. Arrows indicate primers for the verification of the deletion via PCR, which are shown as black lines. The thick grey lines indicate DNA fragments used as probe for Southern hybridization. The restriction sites of the enzymes are displayed, which were used for restriction of the DNA for Southern blot analysis. Dotted lines display areas for homologous integration. (B) PCR analysis for the verification of the *gul1*- and *pro45* deletion. Integration of 5'-flank *gul1*, 3'-flank *gul1* and *gul1* was tested, as well as 5'-flank *pro45*, 3'-flank *pro45* and *pro45* in S156228. Genomic DNA of the wt served as control. Negative control (NK) contained no DNA. (C) Autoradiograph of Southern blot hybridization with radioactively labeled probes specific for *hph*, *gul1* and *pro45*. Genomic DNA for hybridization with *gul1*, *pro45 and hph* was digested with *Hin*dIII, *Eco*RI and *Pvu*II, respectively.
