# Supplementary material for: The STRIPAK signaling complex regulates dephosphorylation of GUL1, an RNA-binding protein that shuttles on endosomes
Source: PLoS Genet. 2020 Sep 30;16(9):e1008819. doi: 10.1371/journal.pgen.1008819 (PMC7550108; doi:10.1371/journal.pgen.1008819)
Supplement: S10 Fig — Lowercase and capital letters indicate the coding sequence of gul1 and the derived amino acid sequence, respectively, close to serine phosphorylation sites S180, S216 and S1343. The triplets encoding the phosphorylated amino acids are given in bold letters and highlighted in grey. Red letters indicate single base pair substitutions and the corresponding amino acid substitutions S180A, S180E, S216A, S216E. S1343A and S1343E. (PDF) [file pgen.1008819.s010.pdf]

	S180	S216	S1343
gul1 wt	/agaagacat**tcc**ttggcc/<br>R R H S L A	/tccgagaag**tct**gaggat/<br>S E K S E D	/**ctctccaagagcccaccg**/<br>L S K S P P
gul1 S180A	/agaagacat**gct**ttggcc/<br>R R H A L A	/tccgagaag**tct**gaggat/<br>S E K S E D	/**ctctccaagagcccaccg**/<br>L S K S P P
gul1 S180E	/agaagacat**gag**ttggcc/<br>R R H E L A	/tccgagaag**tct**gaggat/<br>S E K S E D	/**ctctccaagagcccaccg**/<br>L S K S P P
gul1 S216A	/agaagacat**tcc**ttggcc/<br>R R H S L A	/tccgagaag**gcc**gaggat/<br>S E K A E D	/**ctctccaagagcccaccg**/<br>L S K S P P
gul1 S216E	/agaagacat**tcc**ttggcc/<br>R R H S L A	/tccgagaag**gag**gaggat/<br>S E K E E D	/**ctctccaagagcccaccg**/<br>L S K S P P
gul1 S1343A	/agaagacat**tcc**ttggcc/<br>R R H S L A	/tccgagaag**tct**gaggat/<br>S E K S E D	/**ctctccaaggcccaccg**/<br>L S K A P P
gul1 S1343E	/agaagacat**tcc**ttggcc/<br>R R H S L A	/tccgagaag**tct**gaggat/<br>S E K S E D	/ctctccaaggagccaccg/<br>L S K E P P

S10 Fig. Codonvariants for expression of phosphomimetic and phosphodeficient phosphorylation sites of *gul1*.
