# Supplementary material for: The STRIPAK signaling complex regulates dephosphorylation of GUL1, an RNA-binding protein that shuttles on endosomes
Source: PLoS Genet. 2020 Sep 30;16(9):e1008819. doi: 10.1371/journal.pgen.1008819 (PMC7550108; doi:10.1371/journal.pgen.1008819)
Supplement: S1 Table — The phosphoproteomic study of Δpp2Ac1, Δpro11, and Δpro22 compared to the wild type identified seven phosphorylation sites in GUL1. None is differentetially phosphorylated in the three STRIPAK single deletion strains. For each phosphorylation site of GUL1, log2 ratio of reporter ion intensity in deletion strain and wild type relative to the respective standard deviation is given. Bold numbers indicate an upregulation of the phosphorylation site compared to the wild type. Regular numbers indicate no regulation of the phosphorylation site compared to the wild type. Standard deviations of the ratio of phosphopeptides in mutants compared to wild type: Δpp2Ac1: 0.63; Δpro11: 0.61; pro22: 0.49. (PDF) [file pgen.1008819.s011.pdf]

S1 Table. Identified phosphorylation sites of GUL1 in [1]. The phosphoproteomic study of Δpp2Ac1, Δpro11, and Δpro22 compared to the wild type identified seven phosphorylation sites in GUL1. None is differentetially phosphorylated in the three STRIPAK single deletion strains. For each phosphorylation site of GUL1, log2 ratio of reporter ion intensity in deletion strain and wild type relative to the respective standard deviation is given. Bold numbers indicate an upregulation of the phosphorylation site compared to the wild type. Regular numbers indicate no regulation of the phosphorylation site compared to the wild type. Standard deviations of the ratio of phosphopeptides in mutants compared to wild type: Δpp2Ac1: 0.63; Δpro11: 0.61; pro22: 0.49.

	Phosphosites									
	S180	S210	S216	S510	S1198	T1287	S1289	S1291	T1298	S1343
**Δpp2Ac1/**WT	0.78<br>0.14	/	/	0.46<br>0.56	/	0.63	1.08<br>1.05	0.43<br>0.43	1.02<br>0.76	0.79
**Δpro11/**WT	**1.81**<br>0.57	/	/	**1.33**<br>**1.76**	/	**1.50**	0.78<br>0.77	0.35<br>0.02	1.10<br>0.31	0.54
**Δpro22/**WT	**1.25**<br>0.19	/	/	0.75<br>0.56	/	0.93	1.30<br>1.00	0.63<br>0.24	1.02<br>0.54	0.59

1. Märker R, Blank-Landeshammer B, Beier-Rosberger A, Sickmann A, Kück U. Phosphoproteomic analysis of STRIPAK mutants identifies a conserved serine phosphorylation site in PAK kinase CLA4 to be important in fungal sexual development and polarized growth. Mol Microbiol. 2020.
