# Supplementary material for: The STRIPAK signaling complex regulates dephosphorylation of GUL1, an RNA-binding protein that shuttles on endosomes
Source: PLoS Genet. 2020 Sep 30;16(9):e1008819. doi: 10.1371/journal.pgen.1008819 (PMC7550108; doi:10.1371/journal.pgen.1008819)
Supplement: S2 Table — (PDF) [file pgen.1008819.s012.pdf]

S2 Table. Values for vegetative growth rates of single and double mutant strains to evaluate genetic interactions.

**Strain**	**Vegetative growth rate in mm per day**	**Relative growth rate**
Wild type	19.333±3.535	1
Δpro45	9.555±2.297	0.494
Δpro11	8.000±2.062	0.413
Δgul1	3.667±0.707	0.190
Expected Δpro45Δpro11	0.204	0.011
Δpro45Δpro11	8.555±2.603	0.442
Expected Δpro45Δgul11	0.093	0.005
Δpro45Δgul1	1.333±0.726	0.069
