# Supplementary material for: The STRIPAK signaling complex regulates dephosphorylation of GUL1, an RNA-binding protein that shuttles on endosomes
Source: PLoS Genet. 2020 Sep 30;16(9):e1008819. doi: 10.1371/journal.pgen.1008819 (PMC7550108; doi:10.1371/journal.pgen.1008819)
Supplement: S3 Table — (PDF) [file pgen.1008819.s013.pdf]

S3 Table. Strains used in this work

**Strain**	**Relevant genotype**	**Relevant phenotype**	**Reference**
R19027	Wild type	fertile	* culture collection
S133143	Wild type	fertile	* culture collection
S70823	*fus*	fertile, brown ascospores	[1]
S151468	Δ*ku70::nat*^{r}	fertile	[2]
N161	Δ*pro45::hyg*^{r}	sterile	[3]
S5.7	Δ*pro11::hyg*^{r}	sterile	[4]
S142975	Δpro11::hyg^{r} /Δpro22::hyg^{r}	sterile	This work
S123458	Δpp2Ac1::hyg^{r} /Δpro22::hyg^{r}	sterile	[5]
S156228	Δ*gul1::hyg*^{r} /Δ*pro45::hyg*^{r}	sterile	This work
TVS1A3S66	Δ*gul1::hyg*^{r}*/fus*	fertile	This work
TVS30C1S28	Δ*gul1::*hyg:*:gpd(p)::gul1-gfp::trpC(t)::nat/fus*^{r}	fertile	This work
TVS35D1ESI25	Δ*gul1::hyg*^{r}*::gpd(p)::gfp-gul1::trpC(t)::nat*^{r}*/fus*	fertile	This work
RLS1141	*h2a-mrfp, fus*	fertile	*culture collection
S180E (TVS48C6S1)	Δ*gul1::hyg*^{r}*::gpd(p)::gul1*^{S180E}*-gfp::trpC(t)::nat/fus*^{r}	fertile	This work
S180E (TVS48C6S2)	Δ*gul1::hyg*$^{r::}$*gpd(p)::gul1*^{S180E}*-gfp::trpC(t)::nat*	fertile	This work
S180E (TVS48C3S17)	Δ*gul1::hyg*^{r}*::gpd(p)::gul1*^{S180E}*-gfp::trpC(t)::nat*^{r}*/fus*	fertile	This work
S180A (TVS63A1)	Δ*gul1::hyg*^{r}*::gpd(p)::gul1*^{S180A}*-gfp::trpC(t)::nat*^{r}*/fus*	fertile	This work
S180A (TVS63B1)	Δ*gul1::hyg*^{r}*::gpd(p)::gul1*^{S180A}*-gfp::trpC(t)::nat*^{r}	fertile	This work
S180A (TVS73C1)	Δ*gul1::hyg*^{r}*::gpd(p)::gul1*^{S180A}*-gfp::trpC(t)::nat*^{r}*/fus*	fertile	This work
S216A (TVS64.1C1)	Δ*gul1::hyg*$^{r::}$*gpd(p)::gul1*^{S216A}*-gfp::trpC(t)::nat*^{r}*/fus*	sterile	This work
S216A (TVS64.1B4)	Δ*gul1::hyg*^{r}*::gpd(p)::gul1*^{S216A}*-gfp::trpC(t)::nat/fus*^{r}	sterile	This work
S216A (TVS64.1B4S1)	Δ*gul1::hyg*^{r}*::gpd(p)::gul1*^{S216A}*-gfp::trpC(t)::nat*^{r}	sterile	This work

S216A (TVS64.2A1)	Δgul1::hyg^{r}::gpd(p)::gul1^{S216A}-gfp::trpC(t)::nat^{r}/fus	sterile	This work
S216E (TVS69A5)	Δgul1::hyg^{r}::gpd(p)::gul1^{S216E}-gfp::trpC(t)::nat/fus ^{r}	fertile	This work
S216E (TVS69A6)	Δgul1::hyg^{r}::gpd(p)::gul1^{S216E}-gfp::trpC(t)::nat^{r}/fus	fertile	This work
S216E (TVS69D9)	Δgul1::hyg^{r}::gpd(p)::gul1^{S216E}-gfp::trpC(t)::nat^{r}/fus	fertile	This work
S216E (TVS69D9S1)	Δgul1::hyg^{r}::gpd(p)::gul1^{S216E}-gfp::trpC(t)::nat^{r}/fus	fertile	This work
S1343A (TVS81D3S2)	Δgul1::hyg^{r}::gpd(p)::gul1^{1343A}-gfp::trpC(t)::nat^{r}/fus	fertile	This work
S1343A (TVS83C10S1)	Δgul1::hyg^{r}::gpd(p)::gul1^{1343A}-gfp::trpC(t)::nat^{r}/fus	fertile	This work
S1343E (TVS79B4S1)	Δgul1::hyg^{r}::gpd(p)::gul1^{134E}-gfp::trpC(t)::nat^{r}/fus	fertile	This work
S1343A (TVS80A3S1)	Δgul1::hyg^{r}::gpd(p)::gul1^{1343E}-gfp::trpC(t)::nat^{r}/fus	fertile	This work
TVS74A1	Δgul1::hyg^{r}::gpd(p)::gul1-DsRed::trpC(t)::nat^{r}	fertile	This work
ER132	Wt::hyg^{r}::Tub(p)::gfp::rab5::Tub(t))	fertile	Pöggeler, Göttingen
ER134	Wt::hyg^{r}::Tub(p)::gfp::rab7::Tub(t)	fertile	Pöggeler, Göttingen
TVS94A1	Δgul1::hyg^{r}::gpd(p)::gul1-DsRed::trpC(t)::nat^{r}:: Tub2(p)::egfp::rab7::Tub2(t)::nat^{r}	fertile	This work
TVS96A1	Δgul1::hyg^{r}::gpd(p)::gul1-DsRed::trpC(t)::nat^{r}:: Tub2(p)::egfp::rab5::Tub2(t)::nat^{r}	fertile	This work
S129706	Δpro11::hyg^{r} /Δpro45::hyg^{r}	sterile	This work
S141923	Δpro11::hyg^{r}/fus	sterile	* culture collection
R14785	Wt::nat^{r}::gpd(p)::gfp::pab1::trpC(t)	fertile	This work

*Culture collection of the Department for General and Molecular Botany, Ruhr-University Bochum

nat^{r}: nourseothricin resistance gene; *hph*^{r}: hygromycin B resistance gene*; rpC(t): trpC* terminator from *Aspergillus nidulans, gpd(p): gpd* promotor from *Aspergillus nidulans,* Tub(p): α-tubulin promoter from *Zymoseptoria tritici,* Tub(t): α-tubulin terminator from *Zymoseptoria tritici*

1. Nowrousian M, Teichert I, Masloff S, Kück U. Whole-genome sequencing of *Sordaria macrospora* mutants identifies developmental genes. G3 (Bethesda). 2012;2(2):261-70.
2. Pöggeler S, Kück U. Highly efficient generation of signal transduction knockout mutants using a fungal strain deficient in the mammalian *ku70* ortholog. Gene. 2006;378:1-10.
3. Nordzieke S, Zobel T, Franzel B, Wolters DA, Kück U, Teichert I. A fungal sarcolemmal membrane-associated protein (SLMAP) homolog plays a fundamental role in development and localizes to the nuclear envelope, endoplasmic reticulum, and mitochondria. Eukaryot Cell. 2015;14(4):345-58.
4. Bloemendal S, Bernhards Y, Bartho K, Dettmann A, Voigt O, Teichert I, et al. A homologue of the human STRIPAK complex controls sexual development in fungi. Mol Microbiol. 2012;84(2):310-23.
5. Beier A, Teichert I, Krisp C, Wolters DA, Kück U. Catalytic subunit 1 of protein phosphatase 2A is a subunit of the STRIPAK complex and governs fungal sexual development. mBio. 2016;7(3):e00870-16.
