# Supplementary material for: The STRIPAK signaling complex regulates dephosphorylation of GUL1, an RNA-binding protein that shuttles on endosomes
Source: PLoS Genet. 2020 Sep 30;16(9):e1008819. doi: 10.1371/journal.pgen.1008819 (PMC7550108; doi:10.1371/journal.pgen.1008819)
Supplement: S4 Table — (PDF) [file pgen.1008819.s014.pdf]

S4 Table. Plasmids used in this work

Plasmid	Characteristics	Reference
pDS23	*gpd*(*p*)::*gfp*::*trpC*(*t*), *trpC*(*p*)::*nat*, *URA3*, *bla*	[1]
pRS426	*URA3*, *lacZ*, *bla*	[2]
pSF27-34	*trpC(p)::hph*	[3]
pKO-SMAC07544	1 kb 5’ flank region and 998bp 3’ flank region of *gul1* with trpC(p)::hph in pRS426	This work
p07544-OEC	*gpd(p)::gul1::gfp::trpC(t)* in pDS23	This work
p07544-OEC_S180A	P07544-OEC carrying *gul1*^{S180A} mutation	This work
p07544-OEC_S180E	P07544-OEC carrying *gul1*^{S180E} mutation	This work
p07544-OEC_S216A	P07544-OEC carrying *gul1*^{S216A} mutation	This work
p07544-OEC_S216E	P07544-OEC carrying *gul1*^{S216E} mutation	This work
p07544-OEC_S1343A	P07544-OEC carrying *gul1*^{S1313A} mutation	This work
p07544-OEC_S1343E	P07544-OEC carrying *gul1*^{S1313E} mutation	This work
pDS23-gul1-DsRed	*gpd(p)::gul1::DsRed::trpC(t)* in pDS23	This work
pRSnatRab5	*Tub2(p)::egfp::rab5::Tub2(t)::nat*^{r}	Pöggeler, Göttingen
pRSnatRab7	*Tub2(p)::egfp::rab7::Tub2(t)::nat*^{r}	Pöggeler, Göttingen

1. Schindler D, Nowrousian M. The polyketide synthase gene *pks4* is essential for sexual development and regulates fruiting body morphology in *Sordaria macrospora*. Fungal Genet Biol. 2014;68:48-59.
2. Christianson TW, Sikorski RS, Dante M, Shero JH, Hieter P. Multifunctional yeast high-copy-number shuttle vectors. Gene. 1992;110(1):119-22.
3. Nowrousian M, Cebula P. The gene for a lectin-like protein is transcriptionally activated during sexual development, but is not essential for fruiting body formation in the filamentous fungus *Sordaria macrospora*. BMC Microbiol. 2005;5:64.
