# Supplementary material for: The STRIPAK signaling complex regulates dephosphorylation of GUL1, an RNA-binding protein that shuttles on endosomes
Source: PLoS Genet. 2020 Sep 30;16(9):e1008819. doi: 10.1371/journal.pgen.1008819 (PMC7550108; doi:10.1371/journal.pgen.1008819)
Supplement: S5 Table — (PDF) [file pgen.1008819.s015.pdf]

S5 Table. Oligonucleotides used in this work

Oligonucleotide	Sequence (5' – 3')	Specificity
07544-5-fw	GTAACGCCAGGGTTTTCCCAGTCACGACGGGATCCGCTGTCTGGAGCGAGTAGTCGCTGC	*gul1* 5´ flank with overlap to pRS426
07544-5-rv	CGAGGGCAAAGGAATAGGGTTCCGTTGAGGGATGCAGGAGTTTGCTGGGACGGAC	*gul1* 5´ flank with *hph* overlap
07544-3-fwVS	GCCCAAAAATGCTCCTTCAATATCAGTTGCATGTTTTGATGATTTGGAAAGACTATGCGT	*hph* with *gul1* 3´ flank with overlap
07544-3-rv	GCGGATAACAATTTCACACAGGAAACAGCGGATCCTTGCGTGCGCTCGGGTATCTCCATC	*gul1* 3´ flank with overlap to pRS426
Vor5´SMAC_07544-fw	TTCATTGGGTAGTTGTAAGAG	*gul1* 5´ flank
5´07544-rv	ACTAAAGCGTGTGACGAACG	*gul1* 5´ flank
3´07544-fw	AGATTGCTATGGGGTGTGTC	*gul1* 5´ flank
Vor3´SMAC_07544-rv	CATCGTCGAGTCTTGACGA	*gul1* 3´ flank
SMAC_07544-fw	ATCAGTCGGCCATTCCAAAC	*gul1* start
SMAC_07544-rv	GGAAGCAGGCCGTCCTGGGT	*gul1* end
Vor5´07544_ 2	ACACGGTGTGTCGCGCAC	*gul1* 5´ flank
07544-fwOEC_VS	AGCTTGACTAACAGCTACAGATCTAATGGACCAGCAACAGCAGCC	*gul1, gfp*
07544-rvOEC_VS	ACAGCTCCTCGCCCTTGCTCACCATGAGGGCATAGGGGTTGAGCG	*gul1, gfp*
07544-fwOEN_VS	TCTCGGCATGGACGAGCTGTACAAGATGGACCAGCAACAGCAGCC	*gfp, gul1*
07544-rvOEN_VS	TGATGATTTCAGTAACGTTAAGTGGATTAGAGGGCATAGGGGTTGAGCGAGCGGAT	*gfp, gul1*
07544_fw-Seq	TTCGCGTGAACAAGAAG	*gul1*
07544_fw-Seq2	TGCCTGATGGTAAGATC	*gul1*
Q5_S180A_SMAC_07544_fw_2	GAGAAGACATGCTTTGGCCCTGGCC	*gul1* S180A Codon
Q5_S180A_SMAC_07544_rv_2	TGATGGCCACCGCCGGAA	*gul1* S180A Codon
Q5_S180E_SMAC_07544_fw	GAGAAGACATGAGTTGGCCCTGGCCGATGCG	*gul1* S180E Codon
Q5_S216A_SMAC_07544_fw	CTCCGAGAAGGCCGAGGATGATGCCAAG	*gul1* S216A Codon

Q5_S216AE_SMAC_07544_rv	GAGCCCGAGGCAGGAGCA	*gul1* S216 Codon
Q5_S216E_SMAC_07544_fw	CTCCGAGAAGGAGGAGGATGATGCCAAGACCACC	*gul1* S216E Codon
Q5_S1343A_SMAC_07544_fw	CCAAGGCCCCACCGTAAGTTATATATCCC	*gul1* S1343A Codon
Q5_S1343AE_SMAC_07544_rv	AGAGGTCGGTCTTGAGAATGACAGGC	*gul1* S1343 Codon
Q5_S1343E_SMAC_07544_fw	CCAAGGAGCCACCGTAAGTTATATATCC	*gul1* S1343E Codon
hph1MN	CGATGGCTGTGTAGAAGTACTCGC	*hph*
hph2MN	ATCCGCCTGGACGACTAAACCAA	*hph*
1224KOvp1neu	GAGGTAAGAGCAAGCTTGTC	5´ flank of *pro45*
1224KOvp2neu	GTACCAATGGCAAGATACGC	3´ flank of *pro45*
SMAC_1224 int fw	CCCTGTCACGACTGAACATATTG	*pro45*
1751	GCCATATTTTCCTGCTCTCC	*gpd(p)* forward
1757	AGCTGACATCGACACCAACG	*trpC(t)* reverse
gul1-HR_fw	GCTTGACTAACAGCTACAGATCTATATGGACCAGCAACAGCAGCCTCAAGG	*gul1*
gul1-DsRed-HR_rv	TGATGACGTCCTCGGAGGAGGCCATGAGGGCATAGGGGTTGAGCGAG	*gul1* and *DsRed*
gul1-DsRed-HR_fw	CCGCTCGCTCAACCCCTATGCCCTCATGGCCTCCTCCGAGGACGTCATCA	*gul1* and *DsRed*
DsRed-Ds23-HR_rv	TCCACTAGTTCTAGAGCGGCCGCTCTACAGGAACAGGTGGTGGCGGCCC	pRSDs23 and *DsRed*
